# Impacted Palatal Canines and Diode Laser Surgery: A Case Report

**DOI:** 10.1155/2022/3973382

**Published:** 2022-10-06

**Authors:** Marina Consuelo Vitale, Maria Gloria Nardi, Matteo Pellegrini, Francesco Spadari, Federica Pulicari, Roberto Alcozer, Martina Minardi, Maria Francesca Sfondrini, Karin Bertino, Andrea Scribante

**Affiliations:** ^1^Unit of Orthodontics and Pediatric Dentistry, Section of Dentistry, Department of Clinical, Surgical, Diagnostic and Pediatric Sciences, University of Pavia, Pavia 27100, Italy; ^2^Section of Dentistry, Department of Clinical, Surgical, Diagnostic and Pediatric Sciences, University of Pavia, Pavia 27100, Italy; ^3^Maxillo-Facial and Odontostomatology Unit, Fondazione IRCCS Cà Granda Ospedale Maggiore Policlinico, Milan 20122, Italy; ^4^Department of Biomedical, Surgical and Dental Sciences, University of Milan, Via Della Commenda 10, Milan 20122, Italy

## Abstract

**Introduction:**

Maxillary canine is the most frequent dental element that could likely remain impacted in the bone structure, with a percentage between 1 and 5%. This study presents a case report using a diode laser for surgical-orthodontics disinclusion of a palatal mucosal impacted permanent left upper canine (2.3) and the simultaneous application of an orthodontic bracket.

**Methods:**

After cementation of the trans-palatal bar to the upper first molars with a hook for orthodontic traction, local anaesthesia with articaine was performed, followed by surgical operculectomy using a diode laser (810 nm wavelength, continuous wave mode with a power output of 3 W, and a 0.4 mm diameter optical fiber), and the orthodontic bracket with a passive metal looped ligature was applied. Subsequently, active elastic traction was applied on 2.3 and the upper arch was bonded for the application of a series of orthodontic wires, lace-back, and metal ligatures. A progressive reactivation of the elastic traction and extraction of 6.3 was necessary to translate the canine into the correct arch position.

**Results:**

Diode laser surgical-orthodontic disinclusion of impacted upper canine was performed successively, resulting in a dry surgical field ideal for orthodontic bracket adhesion. No pain and no swelling have been reported from the patient. The orthodontic treatment allowed the canine to be moved to the correct position in the arch.

**Conclusions:**

This case showed that the diode laser is a valid alternative for the surgical-orthodontic disinclusion of an included tooth element.

## 1. Introduction

After the third molar, maxillary canine is the most frequent dental element that could likely remain impacted in the bone structure, with a percentage between 1 and 5%, depending on the patient. It is more common in females than in males, with a ratio of 2 : 1, and only 8% of patients are bilateral [1]. Impaction of the maxillary canine has a ratio of 10 : 1 to mandibular canine and 85% of the upper impaction are palatal [2].

Numerous etiological factors are associated with impacted canines and, according to the literature, they can be classified as localized, systemic, and genetic (Table 1) [3–9].

It is essential to make an early diagnosis of the impacted canine to plan the treatment of this dental anomaly in advance and to properly replace this tooth in the arch as soon as possible, considering the importance of canine guidance role in the occlusion and consequently in the correct body posture. [10]. Early diagnosis of canine displacement in relation to the surrounding structures is mainly based on radiographic examination: in Panoramic X-ray more mesial is located in the canine crown, further likelihood of canine eruption is reduced [11].

The choice of treatment in the patient with an impacted canine changes with the age, oral health, and etiologic factors [3, 12]: monitoring of the impacted element, in the absence of clinical problems [13], extraction of the deciduous element, if the impacted permanent canine is close to eruption [3], extraction of the deciduous tooth and simultaneous uncovering of the permanent tooth with the application of an orthodontic appliance to increase the space in the arch, and allowing the physiological eruption of the canine [13]. In some cases, it is possible to perform dental reimplantation, when the orthodontic treatment turns out to be too complex, unsuccessful, or not accepted by the patient [13].

At the end of growth, surgical-orthodontic treatment is the only choice to align teeth in the dental arch; alternatively, it is possible to extract the impacted element with subsequent prosthetic or implant-prosthetic treatment [3].

Laser is the acronym of light amplification by stimulated emission of radiation, and it represents a new technology introduced in Dentistry by Theodore Harold Maiman in 1960, but only in 1989, Nd:YAG laser and CO_2_ laser were commercially available in the dental market [14]. The light emitted is monochromatic, collimated, and coherent [15]. The essential elements of a laser device are a sounding box in which an active medium is stimulated by a pumping process in a population inversion and in a suitable geometry of the optical feedback elements. Depending on the active medium (gas, liquid, or solid), laser radiation could be in the ultraviolet, visible, or in infrared section of the electro-photometer spectrum [16].

Different authors described laser efficacy in the treatment of oral pathologies, carrying out studies on photodynamic therapy [17, 18] and surgical procedures [19, 20]. Laser provides an excellent alternative to conventional surgical techniques for soft tissue [21]. Many authors described the use of laser in surgical treatments, such as gingivectomy, labial, lingual frenectomies, and fiberotomy [22]. Furthermore, laser can prevent aphthae and cold sores, ablate benign and irritant tissue excess, correct lip, and tongue bands [23]. Laser can also be used in endodontics to remove bacteria from infected teeth [24]. Laser plays an important role in the treatment of periodontal disease: it can modulate many inflammatory pathways, improves wound healing by stimulating fibroblast proliferation, it has a positive biomodulatory effect on bone healing, it performs an antimicrobial activity, and could be a reliable adjunctive agent to mechanical debridement in the non-surgical treatment of periodontal disease [25]. Additionally, it is also used to treat dentin hypersensitivity [26]. Laser can also be useful in dental bleaching: studies have shown how it can accelerate the release of free radicals within the bleaching gel to decrease the duration of the treatment [27]. Some authors described how low-level laser therapy and photodynamic therapy after scaling and root planning can improve the therapeutic outcomes in patients affected by chronic periodontitis [28]. In the literature the role of laser in cavity preparation, carious removal, and pulp therapy is also discussed [29]. Meanwhile, laser, safe and effective technology, has become widely used in dentistry. The potential advantages include less or no bleeding during the surgical procedure, reduction, or elimination of the need for local anaesthesia, reduced post-operative pain, and less patient anxiety [30].

Finally, laser can be applied in orthodontics to etch enamel prior to adhesive application and orthodontic brackets [31] and can reduce pain following band application on maxillary first molars for orthodontic treatment [32]. Photobiomodulation (or low-level laser therapy) with laser or LED can also be used to reduce pain intensity during and following lower third molar extraction and to improve symptoms related to Burning Mouth Syndrome through a reduction of the capillary diameter [33, 34].

Since there are few studies assessing laser application's advantages in surgical-orthodontics disinclusion of an impacted canine, the aim of the present study is to present a case report using a diode laser for the disinclusion of an impacted permanent left upper canine (2.3) and the simultaneous application of an orthodontic bracket.

## 2. Case Report

### 2.1. Diagnosis and Etiology

**Figure 1:**
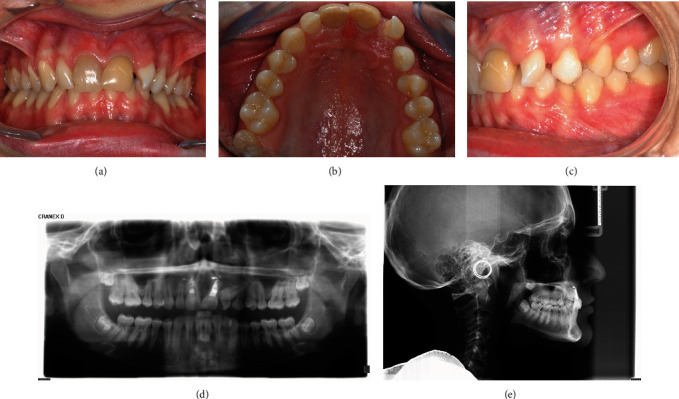
(a–c) Pretreatment intraoral and (d and e) radiographical recordings.

A 17.5-year-old female patient was referred to the Unit of Orthodontics and Paediatric Dentistry from the Unit of Oral Surgery of the Department of Clinical, Surgical, Diagnostic, and Paediatric Sciences of the University of Pavia. In the past, she had a root canal treatment on upper central incisors after dental trauma. The patient showed facial symmetry, competent lips, and normal facial height. Upper and lower dental midlines coincided. Fox plane was not completely parallel to the interpupillary line. Panoramic and lateral cephalometric radiographs were taken (Figures 1(d) and 1(e); Cranex® D, Soredex®, Tuusula, Finland): the panoramic radiograph showed the presence of third molars' germs, lower deciduous canines' persistence, and upper left canine's impaction without apparent left maxillary incisors' radicular resorption.

Cephalometric analysis was performed (DeltaDent, version 2.3, Outside Format, Paullo, Italy): class I skeletal with bimaxillary protrusion (ANB 1.2°, SNA 87°, SNB 85.8°, and Wits index—1.4 mm), hypodivergency, and anterior remaining growth were shown.

The facial profile was normal, the facial angle was slightly increased (171.3°), while the nasolabial angle was slightly decreased (93.3°). Upper incisors were correctly inclined, in fact, the angle formed by the upper incisor's longitudinal axis and bispinal plane (Sna-Snp) measured 107.9°, whereas lower incisors were retroclined, considering that the angle formed by the lower incisor's longitudinal axis and mandibular plane (Go-Gn) measured 87.8°. Intraoral pictures (Figures 1(a)–1(c); taken with Nikon Digital Camera D3500, Nikon Corporation, Tokyo, Japan) showed coincident dental midlines, deep bite, conservative treatments on upper central incisors, and upper lateral incisors' malposition. Overjet measured 0 mm, while overbite measured 4 mm. The patient presented class I molar and class I canine on the right with upper central incisors discolouration. On the left, the patient showed class I molar, while canine classification was not possible. Lower deciduous canines were still present, left maxillary lateral incisors were rotated and many diastemas could be found. The morphology of the upper arch was correct, the retroincisive papilla was hypertrophic, and the first upper maxillary molars were rotated. The lower arch showed a correct morphology as well, lower left canine was lingually inclined. There was no dentoalveolar discrepancy. Bolton's analysis was appropriate, considering that the impacted canine was the same size as the contralateral teeth and excluding the lower left canine. Furthermore, clinical recordings highlighted normal Periodontal Screening and Recording and slight mobility on upper central incisors, multiple gingival recessions in the upper and lower posterior jaws and good oral hygiene.

Clinical and radiological recordings led to the diagnosis of the upper left canine's palatal impaction and surgical exposure of the impacted tooth was planned for subsequent orthodontic traction. The impacted canine provided a “sector 2” position with an *α* angle, formed by the tooth's mid-line and long axis, of approximately 80° and a short distance from the alveolar ridge [35].

### 2.2. Treatment Objectives

The aim of the treatment is surgical operculectomy of the mucosal tissue overlying the left upper permanent canine (2.3) in palatal mucosal impaction, with good surgical haemostasis, allowing intra-operative adhesion of the orthodontic bracket, thus exerting a more rapid onset of orthodontic traction. The extraction of the deciduous canine (6.3) is necessary to allow the permanent canine to be moved into the dental arch after the disinclusion has been completed.

### 2.3. Treatment Alternatives

The first possible alternative is a surgical treatment with lasers of various wavelengths: 10,600 nm (CO_2_ laser), 2940 nm (Er:YAG laser), and 1064 nm (Nd:YAG laser).

Another possible alternative is the use of the electric scalpel, which presents different risks associated with its use, including burns due to the use of high-frequency current, fire risks due to the presence of combustible liquids or gases in the room where it is used, and electrical risks due to the radiated power, which may disturb any equipment applied to the patient [36].

The last alternative is a conventional surgical approach using a cold-blade scalpel, with the consequent risks of intra- and post-operative bleeding and the impossibility of maintaining a dry surgical field, essential to ensure proper adhesion of the orthodontic bracket, the need to apply sutures, potential difficulties in wound healing, and increased post-operative pain [37].

### 2.4. Treatment Progress

**Figure 2:**
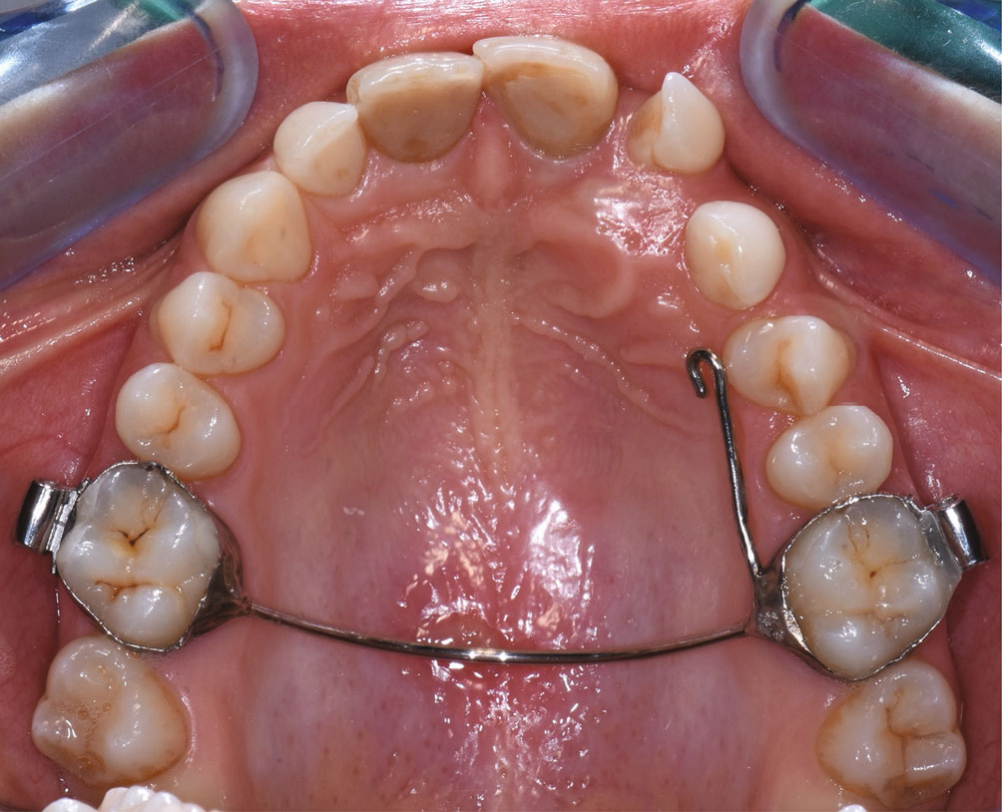
Intraoral photo with the TPB cemented to the first molars and the orthodontic traction hook welded.

**Figure 3:**
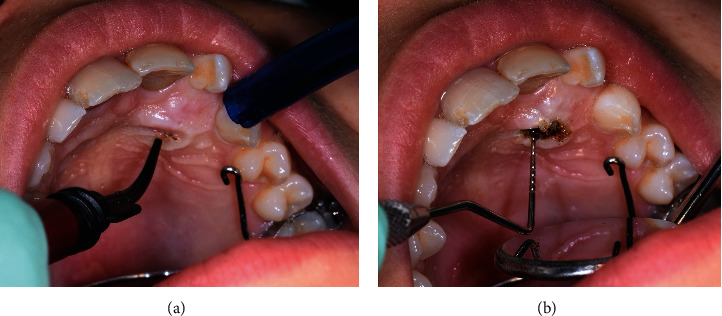
(a and b) Intraoral photo during surgical procedure.

**Figure 4:**
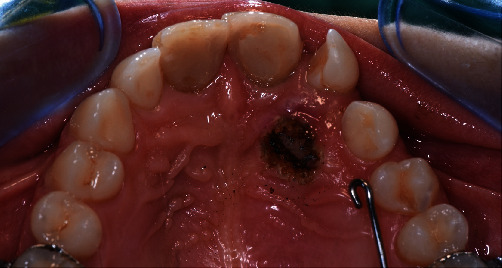
Surgical incision after laser surgery.

Written informed consent was obtained from the patient's parents to proceed with the surgical-orthodontic procedure of canine disinclusion. An upper silicone impression (Elite HD+ Putty Soft, Zhermack S.p.A., Badia Polesine, Italy) was made and sent to the dental technician for fabrication of a trans-palatal bar (TPB) with a hook for orthodontic traction of 2.3 impacted. After the use of orthodontic separators (Loose S Modules S2, 3M Company, St. Paul, MN, USA), the TPB was cemented to the upper first permanent molars (3M™ Unitek™ Multi-Cure Glass Ionomer Band Cement, 3M Company, St. Paul, MN, USA) and cement was light-cured (Starlight Pro, Power Output 1400 mW/cm^2^, Mectron S.p.A., Carasco, Italy; Figure 2). The patient and all staff wore protective glasses to prevent eye damage [38]. After local anaesthesia (Septanest, articaine hydrochloride 4% with adrenaline 1/100,000), a diode laser (Fotona XD-2, Fotona d.o.o., Ljubljana, Slovenia) was used to perform surgical operculectomy, being an element in superficial palatal impaction, with the following parameters [39]: 810 nm wavelength, continuous wave mode with a power output of 3 W and a 0.4 mm diameter optical fiber (Figures 3(a) and 3(b)).  Figure 4 shows how the surgical incision did not cause intra-operative bleeding, thus creating an ideal condition for the adhesion of the orthodontic bracket. No pain and no swelling have been reported from the patient.

The bonding protocol used is as follows [40]: etching of the enamel with 35% phosphoric acid applied for 20–30 seconds and rinsing for an equivalent time. Drying using aspiration to obtain the chalky white appearance of the enamel. Application of light cure adhesive (3M™ Transbond™ PLUS Color Change Adhesive, 3M Company, St. Paul, MN, USA) on the enamel and on the intrados of the orthodontic bracket using a Microbrush® (Microbrush International, Algonquin, IL, USA). Positioning of the orthodontic bracket (3M Unitek APC™ II Victory Series™, 3M Company, St. Paul, MN, USA) and application of continuous pressure for a light curing time of twice 20 seconds under constant suction (Starlight Pro, Power Output 1400 mW/cm^2^, Mectron S.p.A.). Immediate traction with an elastic connection to the wire: if an error has been made in the bonding protocol, the orthodontic bracket is immediately taken off and a new bonding procedure is begun.

**Figure 5:**
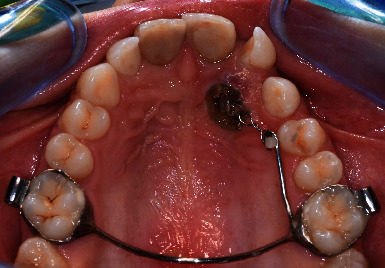
Orthodontic bracket positioned after surgical operculectomy, with a passive metal looped ligature and secured with composite.

The orthodontic bracket was placed with a passive metal looped ligature, secured with composite (Figure 5). No sutures and no additional analgesic or antibiotic were recommended, thanks to the photo-bio-stimulating effect (LLLT) of laser [41]. The patient was discharged with necessary postoperative instructions for maintenance of good oral hygiene and keeping the area clean.

**Figure 6:**
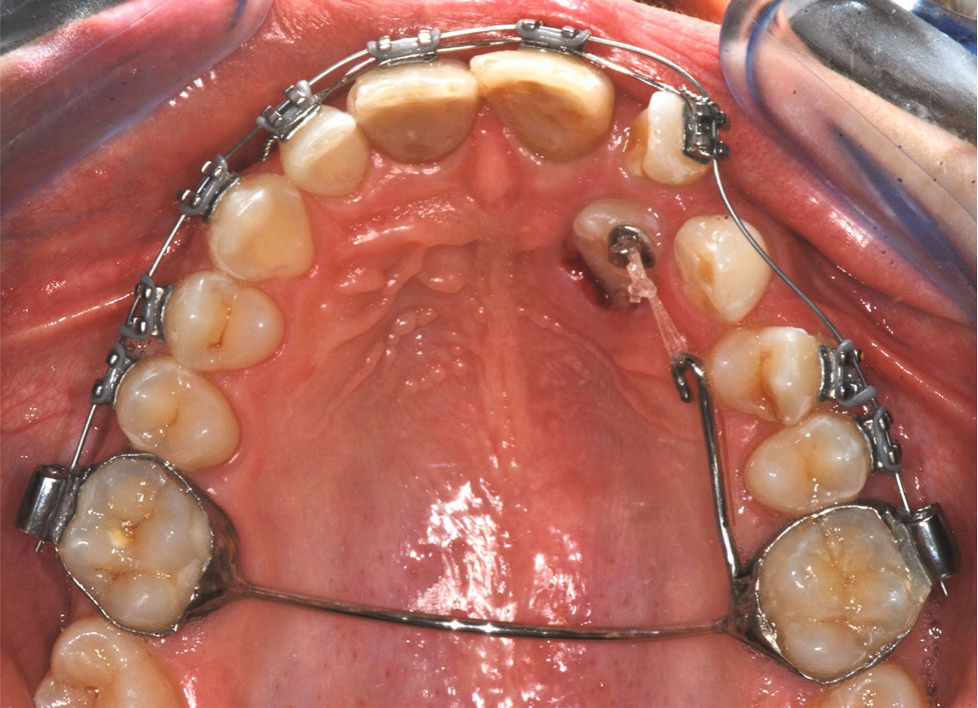
Intraoral photo 1 month after surgery with active elastic traction on 2.3 and upper arch bonded.

The patient was visited with a follow-up of 2 weeks, twice, and monthly thereafter. After 2 weeks, active elastic traction on 2.3 has been applied and one month after surgery the upper arch was bonded through Straight-Wire MBT™ technique with a 0.14″ NiTi arch-wire (3M™ Unitek™ Nitinol Classic Archwire, 3M Company, St. Paul, MN, USA), performing a mesial lace-back 1.2–2.2 and metal ligation on 2.2 with preformed ligature wire 0.10″ (K4210-25, Leone S.p.A. Ortodonzia e Implantologia, Sesto Fiorentino, Italy; Figure 6).

Subsequently, the canine was gradually translated into the correct position in the dental arch by progressive reactivations of elastic traction with elastic cotton threads (Leone, Sesto Fiorentino, Italy), lace-back 1.2–2.2, and metal ligatures on 2.2 and 2.4 with preformed ligature wire 0.10″ (K4210-25, Leone); in addition, a super elastic NiTi arch-wire (0.12″) upper arch has been used (3M™ Unitek™ Nitinol Classic Archwire, 3M Company).

Table 2 shows the procedures checklist of surgical-orthodontic disinclusion of the left upper canine (2.3) in palatal mucosal impaction and its orthodontic translation in the upper arch.

The patient is still undergoing orthodontic treatment to finalize therapy for closing diastema between 2.2 and 2.3.

### 2.5. Treatment Results

**Figure 7:**
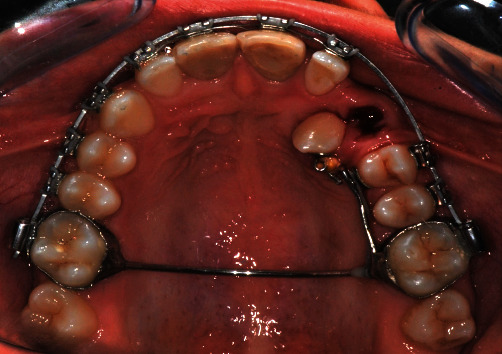
Position of 2.3 after 3 months of orthodontic traction and the extraction of deciduous 6.3.

**Figure 8:**
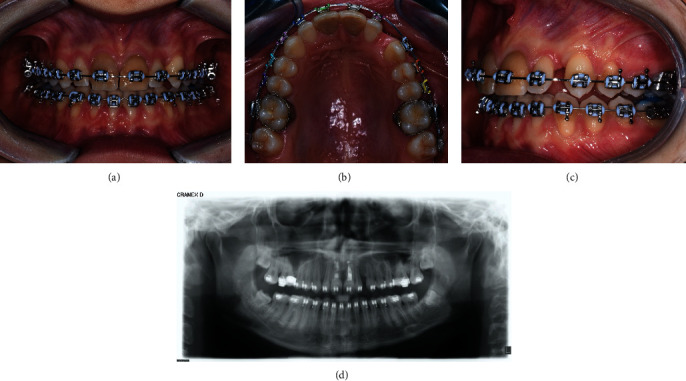
(a–c) Intraoral photo and (d) panoramic radiograph 12 month after surgical-orthodontics disinclusion. Upper canine 2.3, once included, is positioned in the dental arch.

After excision, the patient had no signs of respiratory distress, and no feeding difficulty was reported from the parents. After 2 weeks and 1 month follow-up, the intraoral wound healed without complications and no signs of infection (Figure 6); 3 months after surgery, the position of the upper canine can be appreciated and thanks to the orthodontic traction applied (Figure 7); 12 months after surgery, the upper canine 2.3, once included, is positioned at the level of the dental arch but a diastema remains between 2.2 and 2.3, for which orthodontic finalization is necessary to close it (Figures 8(a)–8(d)).

### 2.6. Discussion

This case report described surgical disinclusion of a left upper canine using a diode laser, followed by orthodontic treatment to translate the impacted canine into the dental arch, highlighting some advantages, such as no intra-operative bleeding, thus providing orthodontic bracket adhesion, no sutures, and no post-operative pain or swelling.

In the literature, there are several articles in which laser technology has been used in the surgical management of impacted teeth; however, to our knowledge, no case reports have not yet been presented in literature where an 810 nm wavelength diode laser has been used to perform surgical operculectomy of the palatal impacted canines with a subsequent intra-operative application of the orthodontic bracket. A previous clinical study showed the application of a 980 nm wavelength diode laser for surgical operculectomy of a palatal included left upper canine, preventing bleeding and need for sutures, reducing pain, and allowing placement of orthodontic brackets [42]. A recent case report showed a surgical operculectomy of both impacted upper permanent canines through a CO_2_ laser, postponing orthodontic bracket placement to a later date and highlighting many advantages, such as no bleeding, no need for sutures, relative ease, and speed of the procedure, reduced or no postoperative symptoms, antimicrobial activity, and rapid wound healing [43]. A case series study showed a combination of two laser (Nd:YAG and Er:YAG) for the surgical treatment of a left upper canine in osteo-mucosal impaction. Er:YAG was used for hard tissue while Nd:YAG for soft tissue. Er:YAG does not show a haemostatic effect, therefore the surgical field was coagulated with Nd:YAG, ensuring bracket adhesion to enamel, and also reducing or avoiding the need for anaesthesia and reducing post-operative pain [44].

In addition, numerous studies in the literature showed the positive effect of the laser on soft tissue, leading to no post-operative pain, no discomfort, no post-operative bleeding, and optimal healing by second intention through the formation of a thin, aesthetically excellent scar. In fact, patients rate the procedure as well tolerable and acceptable [45–47].

A brief review of the dosimetry and techniques used in previous clinical studies is reported in Table 3.

Studies in the literature point to some important disadvantages in the use of the other surgical alternatives, which contributed to the choice of using the diode laser. Specifically, the use of the CO_2_ laser results in thermal damage and a stress effect on soft tissue cells, evident through the presence of elongated cells with fusiform nuclei devoid of nucleoli (therefore inactive), depletion of glycogen, and a reduction in the expression of immunocytochemical markers of intracellular proteins, nuclear cell cycle proliferation, and apoptosis [49]. This cellular damage is also determined following the use of the Er:YAG laser; however, it is significantly reduced, highlighting the possibility of faster healing [49, 50]. An important disadvantage of this laser, considering the importance of having a dry operating field to be able to apply the orthodontic bracket, is the absence of a coagulating effect [49, 50]. Obviously, the same disadvantage is represented using a cold-blade scalpel [37]. The Nd:YAG laser has an unsatisfactory analgesic effect and, consequently, local anaesthesia is required and the fibrous tissue is not easily removed [51]. Finally, the electrosurgical scalpel presents numerous disadvantages and risks associated with its use, from risk of burns, due to the use of high-frequency current, to a fire risk, due to the presence of combustible liquids or gases in the room where it is used, in an electrical risk, due to the radiated power that can disturb various medical devices applied to the patient. Furthermore, it has been shown, from a histological, histochemical, and immunocytochemical point of view, that the epithelial damage due to the use of electrocautery is higher than the epithelial damage induced using the CO_2_ laser, delaying the healing of the surgical wound more [36, 52].

This study has some limitations. First, this is a case report with a short-term follow-up. More cases with long-term follow-up are needed to prove the success of this technique. Finally, we can also consider a limitation: laser equipment can involve a high investment and require a learning curve to achieve an optimal result. Based on these observations, several clinical trials are needed to evaluate the advantages and disadvantages of different laser and electrosurgery in surgical disinclusion of impacted teeth.

## 3. Conclusion

This case report shows how an 810 nm diode laser can be used during orthodontic treatment for dental exposure without causing post-operative pain and swelling and how it allows maintaining a dry field in order to apply an orthodontic bracket during the operation.

## Figures and Tables

**Table 1:** Etiologic factors associated with impacted canines.

Localized [3, 4]				
Loss of arch space	Trauma	Ankylosis	Root dilacerations	Supernumerary teeth
Cyst or neoplasm	Reconstructive surgery for cleft lip/palate repair	Thickened overlying bone or soft tissue	Missing adjacent lateral incisor	Variation in root size of the lateral incisor
Variation in timing of lateral incisor root formation		Over-retained primary canine or early loss of the primary canine		Idiopathic factors
Systemic [5]				
Endocrine disorders		Febrile illness		Irradiation
Genetic [5–9]				
Gardner syndrome	Cleidocranial dysostosis	Yunis–Varon syndrome	Malposed tooth germ	Presence of an alveolar cleft

**Table 2:** Procedures checklist of surgical-orthodontic disinclusion of the left upper canine (2.3) in palatal mucosal impaction and its orthodontic translation in the upper arch.

1. Diagnosis of palatal mucosal impaction of the left upper canine (2.3) by clinical and radiological recordings.
2. Definition of treatment objectives: surgical-orthodontic disinclusion of impacted 2.3 by diode laser and its orthodontic translation in the arch after extraction of 6.3.
3. Obtaining informed consent from the patient's parents to proceed with surgical-orthodontic disinclusion.
4. Performing an upper silicone impression for the fabrication of a trans-palatal bar (TPB) with a hook for orthodontic traction of 2.3 impacted.
5. After application of the orthodontic separators, cementing and light-curing the TPB on the upper first permanent molars.
6. Wearing safety glasses and performing local anaesthesia.
7. Performing a diode laser surgical operculectomy with the following parameters: 810 nm wavelength, continuous wave mode with a power output of 3 W, and a 0.4 mm diameter optical fiber.
8. Adhesion of the orthodontic bracket to achieve orthodontic traction.
9. Etching of the enamel with 35% phosphoric acid applied for 20–30 seconds and rinsing for an equivalent time.
10. Drying using aspiration to obtain the chalky white appearance of the enamel.
11. Application of light cure adhesive on the enamel and on orthodontic bracket baseplate using a Microbrush®.
12. Positioning of the orthodontic bracket and application of continuous pressure for a light curing time of twice 20 seconds under constant suction.
13. Immediate traction with an elastic connected to the wire: if an error has been made in the bonding protocol, the orthodontic bracket is immediately taken off and a new bonding procedure is begun.
14. Placement of the orthodontic bracket with a passive metal looped ligature, secured with composite.
15. Discharge the patient with necessary postoperative instructions for maintenance of good oral hygiene and keeping the area clean.
16. See the patient 2 weeks after surgery, twice, and monthly thereafter. Application of active elastic traction on 2.3 after 2 weeks and upper arch bonding one month after surgery through Straight-Wire MBT™ technique with a 0.14″ NiTi arch-wire, performing a mesial lace-back 1.2–2.2 and metal ligation on 2.2.
17. Progressive translation of the canine into the correct position in the dental arch through reactivations of elastic traction with elastic cotton threads, lace-back 1.2–2.2, metal ligatures on 2.2 and 2.4 and super elastic NiTi (0.12) upper arch.

**Table 3:** Brief review of the dosimetry and techniques used in previous clinical reports.

Authors	Laser	Mode	Power setting	Wavelength (nm)
Migliario et al. [42, 48]	Diode	Pulsed (20 s)	1.5 W	980
Impellizzeri et al. [43]	CO_2_	Superpulsed (80 Hz)	4.5 W	10,600
Fornaini et al. [44]	Nd:YAG	Superpulsed (40 Hz)	4 W	1064
	Er:YAG	Medium-short pulse (10 Hz)	300 mJ	2940
Olivi et al. [45]	Er:YAG	Superpulsed (300 *μ*s)	2.25–3 W	2940
Kato and Wijeyeweera [46]	CO_2_	Continuous	3 W or 4 W	10,600
Ramkumar et al. [47]	Diode	Continuous	1.5 W	940

## Data Availability

The authors confirm that the data supporting the findings of this study are available within the article.
